# Occurrence and molecular characterization of *Cryptosporidium* spp., *Giardia duodenalis*, *Enterocytozoon bieneusi*, and *Blastocystis* sp. in captive wild animals in zoos in Henan, China

**DOI:** 10.1186/s12917-021-03035-0

**Published:** 2021-10-18

**Authors:** Kaihui Zhang, Shuangjian Zheng, Yilin Wang, Ke Wang, Yuexin Wang, Azhar Gazizova, Kelei Han, Fuchang Yu, Yuancai Chen, Longxian Zhang

**Affiliations:** 1 College of Veterinary Medicine, Henan Agricultural University, No. 15 Longzihu University Area, Zhengzhou New District, Zhengzhou, 450046 P. R. China; 2 International Joint Research Center for Animal Immunology of China, Zhengzhou, Henan P. R. China

**Keywords:** *Cryptosporidium* spp., *G. Duodenalis*, *E. Bieneusi*, *Blastocystis* sp., Zoonotic, Zoo

## Abstract

**Background:**

Captive wild animals in zoos infected with *Cryptosporidium* spp., *Giardia duodenalis, Enterocytozoon bieneusi*, and *Blastocystis* sp. can be sources of zoonotic infections and diseases. Therefore, to investigate the distribution of these pathogens in captive wild animals of zoos in Henan, China, a total of 429 fresh fecal samples were collected from six zoos in Henan, China. The infection rates of *Cryptosporidium* spp., *G. duodenalis*, *E. bieneusi*, and *Blastocystis* sp. were determined by PCR analysis of corresponding loci. Positive results for *Cryptosporidium* (*C. parvum* and *C. hominis*) were subtyped based on the (*gp60*) gene.

**Results:**

The overall prevalence was 43.1% (185/429), and the prevalence of *Cryptosporidium*, *Giardia duodenalis, Enterocytozoon bieneusi*, and *Blastocystis* sp. were 2.8% (12/429), 0.5% (2/429), 20.8% (89/429), and 19.1% (82/429), respectively. Five *Cryptosporidium* species, namely, *C. hominis*, *C. parvum*, *C. muris*, *C. andersoni*, and *C. macropodum*, were identified in this study. *Cryptosporidium parvum* was further subtyped as IIdA19G1. Two *Giardia duodenalis* assemblages (A and E) were also identified. A total of 20 *Enterocytozoon bieneusi* genotypes were detected, including 18 known (BEB6, D, HND-1, CD7, SDD1, Henan-IV, KIN-1, CHK1, Peru8, Henan-V, CHG11, CHG-1, CHS9, CHG21, Type-IV, CHC9, CM5, and CHB1) and 2 novel genotypes (CHWD1 and CHPM1). A total of nine subtypes of *Blastocystis* sp. (ST1, ST2, ST3, ST5, ST6, ST7, ST10, ST13, and ST14) were identified in captive wild animals in zoos in the present study. *Cryptosporidium andersoni,* nine *Enterocytozoon bieneusi* genotypes, and five *Blastocystis* subtypes were here first identified in new hosts.

**Conclusions:**

Our study has expanded the host ranges of these four pathogens. The data indicate that animals in zoos can commonly be infected with these four zoonotic pathogens, and animals in zoos are potential sources of zoonotic infections in humans.

**Supplementary Information:**

The online version contains supplementary material available at 10.1186/s12917-021-03035-0.

## Background

*Cryptosporidium* spp., *Giardia duodenalis*, *Enterocytozoon bieneusi,* and *Blastocystis* sp. are four common opportunistic pathogens with wide host ranges that include livestock, wildlife, and humans [1–4]. Infections with these pathogens can cause diarrhea and several other gastrointestinal illnesses in humans and animals [1–4]. The fecal-oral route is the main transmission pathway of the four pathogens, and infection can also result from contaminated food or water [2, 4].

Currently, at least 44 valid species and about 70 genotypes of *Cryptosporidium* have been described, and at least 20 species and 5 genotypes have been detected in humans [4, 5]. *Giardia duodenalis* is considered a species complex with at least eight distinct assemblages (A-H), and assemblages A and B are infectious to humans and other mammals as well as a wide range of hosts [6]. Over 474 *Enterocytozoon bieneusi* genotypes were distributed in several genetically isolated populations comprising 11 major groups in a phylogenetic analysis, including zoonotic group 1 and several host-adapted groups [3, 7]. Among 17 approved subtypes (ST1-ST17) of *Blastocystis* sp., ST1–ST9 and ST12 have been observed in humans. Two subtypes (ST9 and ST12) are specific to humans, and the remaining subtypes have been detected in non-human hosts [8, 9].

For many animal species, life in a zoo is very different from natural free-range conditions. Due to the limitations of living space, the prevalence of parasites in captive animals is often higher than that of wild animals [10]. Animal keepers can be in close contact with animals through feeding, washing, and cleaning, and visitors can indirectly contact animals by petting or by giving food. Previous studies have found *Cryptosporidium* and *Blastocystis* in zoo animals and their keepers [11–13]. There is a potential for zoonotic transmission between animals and humans in zoos [14]. The objectives of this study are to examine the prevalence and determine the genetic distributions of *Cryptosporidium*, *G. duodenalis*, *E. bieneusi*, and *Blastocystis* and to identify genotypes/assemblages of human health importance..

## Results

### Occurrence of *Cryptosporidium*, *Giardia duodenalis*, *Enterocytozoon bieneusi*, and *Blastocystis*

The overall infection rate was 43.1% (185/429, 95% CI: 39.33–48.77%, *χ*^2^ = 25.048, *df* = 5, *P* < 0.001) among six zoos. The prevalence of *Cryptosporidium* spp., *Giardia duodenalis*, *Enterocytozoon bieneusi,* and *Blastocystis* sp. were 2.8% (12/429, 95% CI: 1.23–4.36%, *χ*^2^ = 23.613, *df* = 5, *P* < 0.001), 0.5% (2/429, 95% CI: 0–1.11%, *χ*^2^ = 21.936, *df* = 5, *P* < 0.001), 20.8% (89/429, 95% CI: 16.89–24.59%, *χ*^2^ = 25.877, *df* = 5, *p* < 0.001), and 19.1% (82/429, 95% CI: 16.24–23.85%, *χ*^2^ = 7.696, *df* = 5, *p* > 0.05), respectively (Table 1). Co-infection results showed that 29 samples were infected by two kinds of parasites; the infected species were sika deer (*n* = 6), white kangaroos (*n* = 4), macaques (*n* = 4), black-and-white colobus monkeys (*n* = 3), two giraffes (*n* = 2), a Bactrian camel (*n* = 1), a patas monkey (*n* = 1), a peafowl (*n* = 1), a pony (*n* = 1), a leopard (*n* = 1), a golden sub-nosed monkey (*n* = 1), a white-browed monkey (*n* = 1), a green monkey (*n* = 1), a squirrel monkey (*n* = 1), and a northern pigtail macaque (*n* = 1).

**Table 1:** Occurrence of *Cryptosporidium* spp., *G. duodenalis*, *E. bieneusi*, and *Blastocystis* sp. in this study

Collection site	Sample size	Prevalence (%) (95% CI)				
		*Cryptosporidium* spp.	*G. duodenalis*	*E. bieneusi*	*Blastocystis* sp.	Total
Xinxiang Zoo	23	–	–	13.0 (0.0–30.0)	13.0 (0.0–30.0)	26.1 (6.0–46.2)
Kaifeng Zoo	36	11.1 (0.0–22.8)	5.6 (0.0–14.4)	2.8 (0.0–9.5)	11.1 (0.0–22.8)	30.6 (14.1–47.0)
Luoyang Zoo	27	–	–	18.5 (2.0–35.0)	22.2 (4.7–39.8)	40.8 (20.4–61.1)
Shangqiu Zoo	24	–	–	20.8 (2.5–39.5)	12.5 (0.0–26.0)	33.3 (24.5–42.2)
Swan Lake Zoo	120	6.7 (1.8–11.6)	–	11.7 (5.5–17.8)	15.0 (8.2–21.8)	33.3 (24.5–42.2)
Zhengzhou Zoo	199	–	–	30.7 (24.4–37.3)	25.1 (18.9–31.4)	55.8 (48.6–62.9)
Total	429	2.8 (1.1–4.5)	0.5 (0.0–1.2)	20.8 (16.8–24.7)	19.1 (16.1–24.0)	43.1 (39.2–48.9)

Note: Negative results denoted by hyphen (“-”)

### *Cryptosporidium* species and subtypes

Five *Cryptosporidium* species, namely *C. hominis*, *C. parvum*, *C. andersoni*, *C. muris*, and *C. macropodum* were identified in this study (Table 2). The *Cryptosporidium hominis* and *C. parvum* samples were further subtyped based on *gp60* gene sequence analysis, with all *C. parvum* identified as subtype IIdA19G1. *Cryptosporidium hominis* was not successfully identified. The three *gp60* sequences showed 99.7% nucleotide sequence identity to the isolates from dairy cattle (MF074761) and *Homo sapiens* (JQ796092) from China.

**Table 2:** Distribution of *Cryptosporidium* and *Giardia duodenalis* in this study

Species/Assemblages		Common name (Positive no.) Accession number
*Cryptosporidium* spp.	*C. muris* (*n* = 4)	Bactrian camel (4) MN038146
	*C. parvum* (*n* = 3)	Squirrel monkey (1) MT648440; Pony (1) MT648441; Ostrich (1) MT648442
	*C. andersoni* (*n* = 3)	Whooper Swan (2) MT648437; **South China tiger (1) MT648443**
	*C. hominis* (*n* = 1)	Black-capped capuchins (1) MT648439
	*C. macropodum* (*n* = 1)	**White kangaroo (1)** MT648438
*Giardia duodenalis*	A (*n* = 1)	Bactrian camel (1) MN047217
	E (*n* = 1)	Bactrian camel (1) MN047216

Note: New genotypes or new hosts are indicated in bold

### *Giardia duodenalis* assemblages

Two *Giardia duodenalis* assemblages, A and E were detected based on *SSU* rRNA and *gdh* loci (Table 2). Assemblage A shared 100% similarity with the sequence from Brazilian *Panthera* (HM134217), and Assemblage E was identical to the isolate derived from dairy cattle in China (KF843926).

### *Enterocytozoon bieneusi* genotypes

**Fig. 1:**
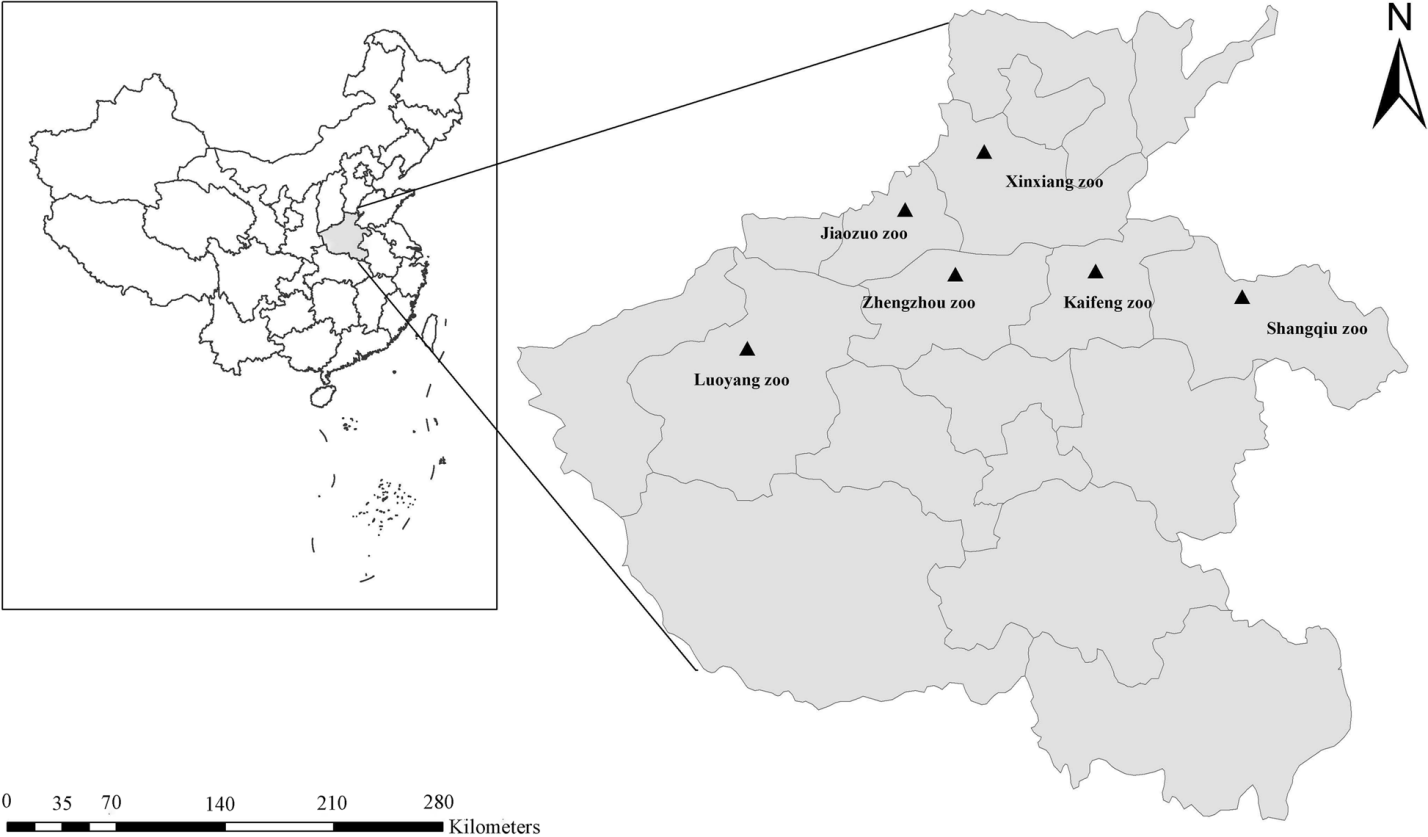
Locations where specimens were collected in this study. The figure was originally designed by the authors using the software ArcGIS 10.2. No copyright permission was required. The original vector diagram imported in ArcGIS was adapted from Natural Earth (http://www.naturalearthdata.com)

A total of 20 genotypes of *Enterocytozoon bieneusi* were identified in the present study, including 18 known genotypes: BEB6, D, HND-1, CD7, SDD1 Henan-IV, KIN-1, CHK1, Peru8, Henan-V, CHG11, CHG-1, CHS9, CHG21, Type-IV, CHC9, CM5, and CHB1. However, a novel genotype CHPM1 was found in a patas monkey, and CHWD1 was found in a white-lipped deer. Additionally, SDD1, BEB6, CD7, HDN-1, CHG-1, CHC9, D, Peru8, and Type-IV were identified for the first time in animal hosts. The most prevalent *E. bieneusi* genotype was BEB6 (32/89, 36.0%) followed by D (16/89, 18.0%) (Table 3). Compared with genotype D (KX383624), novel genotypes CHPM1 and CHWD1 had one and three SNPs based on the ITS region, respectively (Table S2). Phylogenetic analysis of *E. bieneusi* showed that genotypes D, Peru8, SDD1, HND-I, Type-IV, KIN-1, Henan-IV, Henan-V, CHPM1, and CHWD1 were clustered in Group 1, whereas CHG11, CHG-1, BEB6, CM5, CHC9, and CHS9 were clustered into Group 2. CHG21, CD7 and CHB1, CHK1were clustered into Group 9, Group 11 and Group10, respectively (Fig. 1).

**Table 3:** *Enterocytozoon bieneusi* ITS genotypes identified in this study

Genotype	Common name (Positive no.) Accession number
BEB6	**Giraffe (1) MT652649**; **White kangaroo (1) MT652664**; Rhinos (1) MT652665; Rhyticeros (2) MT652666; Macaws (1) MT652667; Yellow-billed Parrot (1) MT652675; Toucan (1) MT652676; Black-necked crane (1) MT652668; **Emu (7) MT652669**; **Elephant (2) MT652670;** Sika deer (5) MT652672; Wildebeest (2) MT652673; Stump-tailed macaque (1) MT652674; Red-crowned crane (2) MT652677; Northern raccoon (1) MT652678; Golden monkey (1) MT652679; Eastern Black-and-white colobus (1) MT652683; Gibbon (1) MT652685
D	**Emu (1) MT652645**; Giraffe (1) MT652647; White lion (2) MT652650; Leopard (1) MT652653; Lion (2) MT652654; **Serval (1) MT652655**; Brown bear (3) MT652657; Siberian tiger (2) MT652661; Golden monkey (1) MT652680; Northern pigtail macaque (1) MT65268; Reeves’s pheasant (1) MT652690
Henan-IV	South China tiger (1) MT652662; White browed monkey (1) MT652686; Green monkey (1) MT652687
KIN-1	White lion (1) MT652651; Black bear (1) MT652656; Squirrel monkey (1) MT652688
CHK1	White kangaroo (3) MT652663
SDD1	**Emu (1) MT652646**; **Macaque (2) MT652659**; **Whooper Swan (1) MT652689**
Peru8	**Emu (1) MT652643**; Eastern Black-and-white Colobus (1) MT652684
CHG11	Giraffe (1) MT652648; Macaque (1) MT652660
Type-IV	**Emu (1) MT652644**
CHG-1	**leopard (1) MT652652**; Peafowl (1) MT652691
CHB1	Brown bear (1) MT652658
CM5	Golden monkey (1) MT652681
CHC9	**Elephant (1) MT652671**
Henan-V	Macaque (2) MT674937
HND-1	**Sike deer (5) MT652692**; **Fallow deer (1) MK931402**
CD7	**White-lipped deer (1) MK931406**; **Sika deer (2) MK931407**; Bactrian camel (3) MK931405
CHS9	Bactrian camel (1) MK931400
CHG21	Eastern Black-and-white colobus (1) MK931399
CHPM1	Patas monkey (1) MK931403
CHWD1	White-lipped deer (1) MK931404

Note: New genotypes or new hosts are indicated in bold

### *Blastocystis* subtypes

A total of nine *Blastocystis* subtypes were found, including ST1, ST2, ST3, ST5, ST6, ST7, ST10, ST13, and ST14. However, the other six subtypes were identified in new hosts for the first time: ST2, ST3, ST5, ST6, ST7, and ST10 were detected in ponies, an orangutan, a gorilla, sika deer, white kangaroos, a blue eared-pheasant, a whooper swan, and giraffes. The most prevalent *Blastocystis* subtype was ST5 (19/86, 22.1%) followed by ST10 (18/86, 20.1%) (Table 4).

**Table 4:** Distribution of *Blastocystis* sp. in the wildlife in this study

Subtypes	Common name (Positive no.) Accession number
ST1	Macaque (6) MT661531; Golden monkey (1) MT661544; Northern pigtail macaque (1) MT661546; Green monkey (2) MT661549; Eastern black-and-white colobus (1) MK930348; Crab-eating macaque (1) MT661550; Japanese macaque (1) MT661551; Northern raccoon (1) MT661552; Squirrel monkey (1) MT661553; White kangaroo (2)
ST2	Chimpanzee (1) MT661543; **Pony (2) MT661555**; Macaque (2) MT661556
ST3	Macaque (3) MT661530; Chimpanzee (1) MT661540; **Orangutan (1) MT661541**; Gorilla (1) MT661542; Patas monkey (2) MT661545; Eastern black-and-white colobus (2) MT661547; White browed monkey (2) MT661548
ST5	Giraffe (1) MT661528; leopard (1) MT661529; **White kangaroo (5) MT661532**; Ostrich (6) MT661533; **Sika deer (6) MT661537**
ST6	Blue-eared pheasant (1) MT661557; Peafowl (3) MT661558
ST7	Turkey (1) MT661534; Whooper Swan (1) MT661554
ST10	**Giraffe (2) MT661527;** Sika deer (12) MT661536; Bactrian camel (1) MT661538; Yak (2) MT661539; White-lipped deer (1) MK930352
ST13	White kangaroo (4) MT672637
ST14	Bactrian camel (1) MK930360

Note: New genotypes or new hosts are indicated in bold

## Discussion

In the present study, the *Cryptosporidium* prevalence was 2.8%, which is lower than the rates (70.0%) reported in Xining Zoo in China [15] and zoo mammals (35.8%) in Barcelona, Spain [16]. However, the infection rate was higher than those in zoos (2.0%) in Alberta and Manitoba of Canada [17] and Zhengzhou Zoo (1.6%) in China [18]. For *Giardia duodenalis*, the prevalence was 0.5%, which is lower than the rate found in most studies conducted in zoos worldwide, for example the 2.5% in Zhengzhou Zoo in China [18], 3.3% in the zoo in Aprilia, Italy [19], and 24.0% in a zoological garden in Poland [20]. The overall infection rate of *E. bieneusi* was 20.8%, lower than in a previous study conducted in the zoo (32.5%) of Howard County in America [21] and higher than in Zhengzhou Zoo (15.8%) in China [18]. Concerning *Blastocystis*, the infection rate was 19.1%, which was higher than the 6.6% found in four zoos in Italy [21] and 12.3% in a zoological garden in Poland [20]. In contrast, the prevalence was lower than that (40.2%) in Qinling Zoo in China [8]. Infection rates may be influenced by many factors, including the zoo management model, living conditions, the immune status of the animals, climate, and geography.

This study demonstrates a high sample prevalence and diversity of intestinal parasites in captive wild animals in zoos in Henan, China. The present study indicated that *Giardia duodenalis* was only found in Kaifeng Zoo, and *Cryptosporidium* spp. was found in Kaifeng Zoo and Swan Lake Zoo. *Enterocytozoon bieneusi* and *Blastocystis* sp. were found in 6 zoos. It can be concluded that *Enterocytozoon bieneusi* and *Blastocystis* sp. are common in some zoos in Henan Province, and among the 6 zoos, the infection rate of *Enterocytozoon bieneusi* is different. The highest infection rate is Zhengzhou Zoo and the lowest is Kaifeng Zoo. The result of *Blastocystis* sp. is consistent with *E. bieneusi*. So the prevalence and diversity varied with geographic region.

Zoonotic *Cryptosporidium* species (*C. hominis, C. parvum, C. muris,* and *C. andersoni*) and *C. macropodum* were identified in the present study. *Cryptosporidium hominis* and *C. parvum* are responsible for most cases of cryptosporidiosis in humans [22, 23]. *Cryptosporidium parvum* subtyping revealed IIdA19G1 in our study that has previously been found in humans, dairy cattle, and yaks in China [24]. Since the first report of *C. muris* in human samples in 2000, evidence of human infection with *C. muris* has been accumulating [25]. Including diarrhea patients cattle, sheep, and the cactus mouse [24, 26], *C. andersoni* was found in a south China tiger in the present study, thereby expanding the host range of *C. andersoni*. *Cryptosporidium macropodum* (only detected in Australia previously) [27] was detected for the first time in a white kangaroo in China.

Similar to previous reports [28], we identified two *G. duodenalis* assemblages (A and E) in the Bactrian camel. Assemblage A was one of the two species of *G. duodenalis* most commonly detected in human samples, and this assemblage has also been detected in livestock, companion animals, and non-human primates (NHPs) [2]. Assemblage E has been reported as an assemblage with host specificity, mainly infecting cattle, sheep, goats, and pigs. However, there are still some studies reporting the presence in human [29]. Therefore, the data indicate that animals in these zoos may serve as reservoirs of *G. duodenalis* assemblages with the potential for zoonotic infection in humans. However, this speculation needs further research and data to confirm.

A total of 20 *Enterocytozoon bieneusi* genotypes were observed in 89 positive specimens. BEB6 was the predominant *E. bieneusi* genotype in the current study; it has been found in NHPs, sheep and goats, companion animals (cats and dogs), chinchillas, rabbits, meerkats, and bats. The genotypes D, type IV, and Peru8 were previously detected in humans, goats, pigs, and NHPs, and they have been frequently found in different water sources, suggesting the likelihood of zoonotic or waterborne transmission [30–33]. Indeed, previous reports have found genotype BEB6, D, type IV, and Peru8 in humans and wildlife in various countries [22, 34, 35]. Therefore, these findings indicate that zoonotic transmission to humans and between wildlife species may occur in the zoos investigated in the present research. We also found the newly identified genotypes CHPM1and CHWD1 in white-lipped deer and patas monkeys, respectively. Interestingly, the isolation of novel genotypes from white-lipped deer, as well as the three novel genotypes identified by the study of Li et al., suggest that genetic variability in deer-derived *E. bieneusi* may be common [3]. Phylogenetic analysis showed that genotypes D, Peru8, SDD1, HND-I, Type-IV, KIN-1, Henan-IV, Henan-V, CHPM1, and CHWD1 were clustered in Group 1, which was composed of zoonotic genotypes. Genotypes CHG11, CHG-1, BEB6, CM5, CHC9, and CHS9 were clustered in Group 2, and other data indicated that the genotypes in Group 2 may have zoonotic potential. Accumulating evidence indicates that some Group 2 genotypes (I, J, BEB4, and BEB6) can also be detected in humans.

A total of nine subtypes were detected in *Blastocystis*, including ST1, ST2, ST3, ST5, ST6, ST7, ST10, ST13, and ST14. Studies indicated that ST1 to ST9 and ST12 have the ability to infect humans [8]. For example, ST1, ST3, and ST5 have been found in NHPs and their keepers, in southern hairy-nosed wombats and their keepers, and in pigs and their workers, respectively [12, 13]. Although the fecal samples of animal keepers were not considered in the present study, the possibility of transmission between animals and animal keepers is undeniable. Therefore, subtypes ST1, ST2, ST3, and ST5 may have potential risk for zoonotic transmission between animals and humans. Present study indicated that the dominant species of *Blastocystis* subtypes in NHPs is ST1-ST3, which is consistent with other results [36–39]. In addition, we found that ostriches can be infected by ST5, a subtype that has been isolated sporadically from many other animals, including NHPs, camels, the black rhinoceros, and rodents [13, 40]. ST6 was found in blue-eared pheasant and peafowl, and ST7 was found in turkeys and the whooper swan, similar to previous findings [36]. ST10 was previously identified in fallow deer and camels [41]. Similarly, ST10 was detected in sika deer and yellow deer in the present study. Only ST14 was found in Bactrian camels here, which is consistent with previous findings [36]. The above results demonstrate that these subtypes can infect animal species in zoos as well as humans, and thus more attention should be paid to these parasites.

Some species, genotypes and subtypes of these parasites were detected in some new hosts in present study. However, what can not be determined is that whether these animals are natural hosts or carriers of theses pathogens and whether these parasites can cause infections in new hosts. Animals could get infected via eating food or drinking water which contain viable pathogens. Considering the specificity of zoo environment, where different species of animals are kept in seperate areas, the risk of cryptosporidiosis transmission through contaminated food or water seems relatively low. Due to the rarity of wild captive animals and the limitation of the amount of pathogen infection, it is difficult for us to demonstrate cross-transmission in different species of wild captive animals. We only successfully infected BALB/c mice with the oocysts of *C. muris* [42]. Efforts should be made in the following study to conduct more investigative research on these problems.

## Conclusion

Our results indicate that animals in zoos can be infected with human pathogenic *Cryptosporidium* spp.*, Giardia duodenalis*, *Enterocytozoon bieneusi,* and *Blastocystis* sp. These animals can serve as reservoirs of human cryptosporidiosis, giardiasis, microsporidiosis, and blastocystosis. Effort should be made to conduct more experimental work to reveal the genetic characteristics and assess the zoonotic risks of these parasites.

## Methods

### Study area and sample collection

**Fig. 2:**
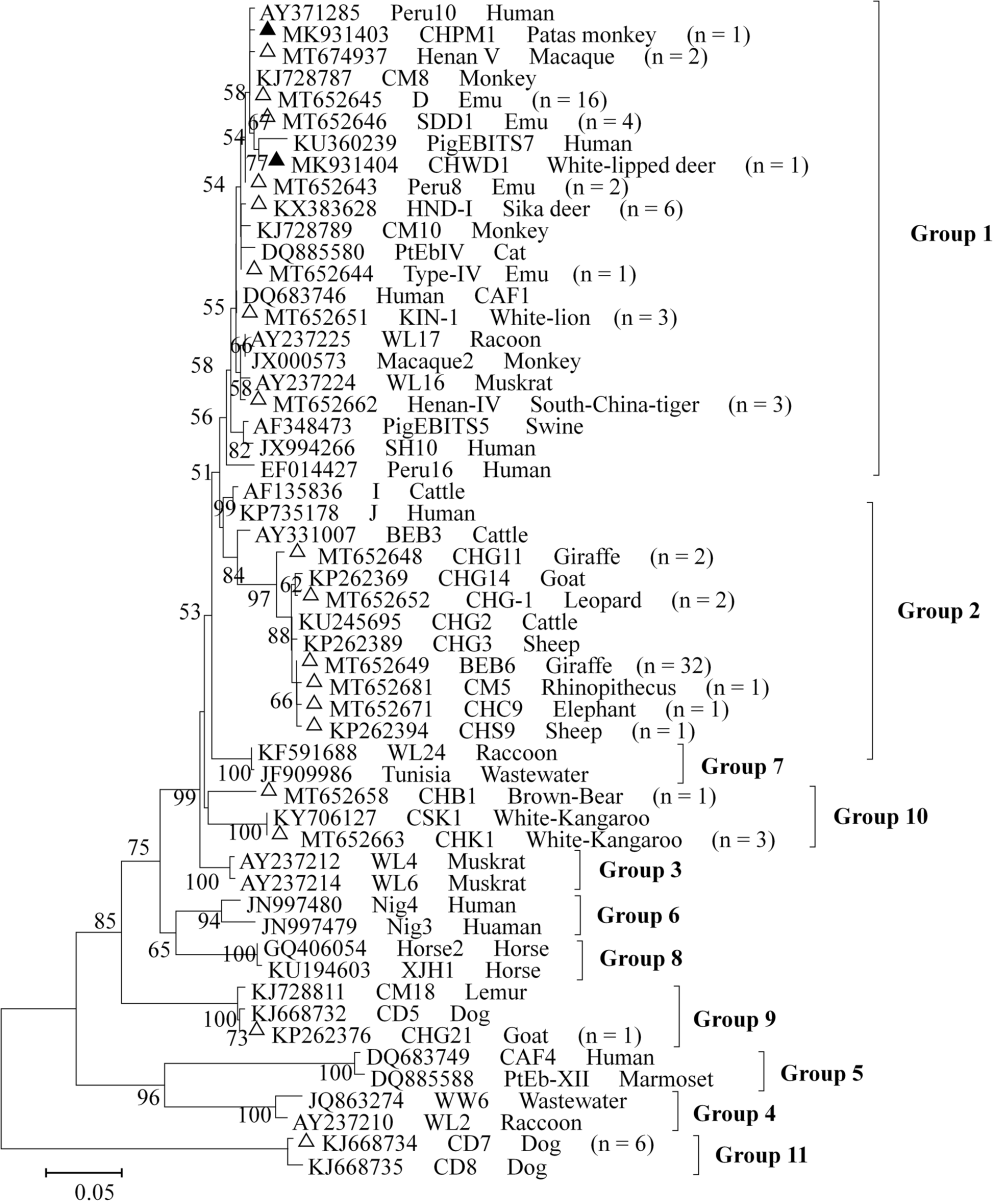
Neighbor-joining tree of *Enterocytozoon bieneusi* ITS genotypes. Phylogenetic relationships of ITS nucleotide sequences of the *Enterocytozoon bieneusi* genotypes identified in this study and other reported genotypes. The phylogeny was inferred by a neighbor-joining analysis. Bootstrap values were obtained using 1000 replicates; those with values > 50% are shown on the nodes. The genotypes in this study are marked by empty triangles, and the novel genotypes are marked by filled triangles

Between October 2018and June 2020, a total of 429 fresh fecal samples (77 animal species) were collected from captive animals in 6 zoos in Henan, China; these were Xinxiang Zoo (*n* = 23), Kaifeng Zoo (*n* = 36), Luoyang Zoo (*n* = 27), Shangqiu Zoo (*n* = 24), Jiaozuo Swan Lake Zoo (*n* = 120) (private zoo), and Zhengzhou Zoo (*n* = 199) (Fig. 2). Some of the specimens tested in this study were animals imported from abroad and were ill (slight or severe diarrhea, and some adult worms in fecal samples) (Table S1). One specimen per animal was used in this study. Only the central portion of the fecal material was collected during sampling to ensure no environmental contamination. Each fresh sample was collected into a sterile glove, labeled, and placed into a container with ice packs and immediately sent to the lab for DNA extraction.

### DNA extraction

200 mg of fecal samples were used to extract DNA with an E.Z.N.A.® Stool DNA Kit (Onmega Biotek Inc., Norcross, GA, USA), according to the manufacturer’s instructions, and the extracted DNA was stored at − 20 °C before PCR analysis. The quality of the DNA extracted was determined by using the NanoDrop absorbance ratio.

### PCR amplification

All of the samples were amplified by nested PCR to identify *Cryptosporidium* spp. and *Giardia duodenalis* based on the small subunit (*SSU*) rRNA gene and the glutamate dehydrogenase (*gdh*) gene [43, 44], respectively. Positives for *Cryptosporidium* (*C. parvum* and *C. hominis*) were subtyped based on the 60-kDa glycoprotein (*gp60*) gene [45]*. Enterocytozoon bieneusi* and *Blastocystis* sp. were identified based on the ITS region [46] and the *SSU* rRNA gene [47], respectively (Table S3). The amplification was performed in 25 μL reaction mixtures. The first reaction mixture contained 1 μL of extracted DNA. The second reaction mixture contained 1 μL of the first PCR amplification product. The KOD Plus DNA polymerase (Toyobo Co., Ltd., Osaka, Japan) was used for all PCR amplification. Positive and negative control samples (distilled water) were included in each PCR assay, and two replicates of each PCR were run for all of the samples. The final PCR products were subjected to 1.0% agarose gel electrophoresis and visualized by staining with DNAGREEN (Tiandz, Inc., Beijing, China).

### Sequencing and phylogenetic analysis

All of the final positive PCR products were sequenced using the ABI PRISM™ 3730 XL DNA Analyzer with the BigDye Terminator v3.1 Cycle Sequencing Kit (Applied Biosystems, Foster City, CA, USA), and two-directional sequencing was used to ensure accuracy. To identify different species or genotypes, sequences obtained were aligned with the reference sequences in GenBank (http://blast.ncbi.nlm.nih.gov) using the software Clustal X 2.1 (http://www.clustal.org/). The phylogenetic relationships of *E. bieneusi* genotypes were analyzed by the neighbor-joining algorithm in MEGA 7.0 (http://www.megasoftware.net/). Bootstrap values were calculated by analyzing 1000 replicates. The established nomenclature system was used in the naming of *E. bieneusi* ITS genotypes [48].

### Statistical analysis

The infection rates with 95% confidence intervals (CI) were calculated by Wald’s method in SPSS 22.0 version (SPSS Inc., Chicago, IL, United States). Differences in corresponding infection rates among locations were examined by the Chi-square test, and differences were considered significant at *P* < 0.05.

## Supplementary Information

**Additional file 1: Table S1.** Specimens from wildlife at six zoos in Henan, China examined in this study.

**Additional file 2: Table S2.** Nucleotide substitutions and indels at the ITS region of CHPM1 and CHDW1 genotypes.

**Additional file 3: Table S3.** Primers and reaction conditions used in the characterization of the SSU rRNA gene of *Cryptosporidium* spp., *Giardia duodenalis*, *Enterocytozoon bieneusi*, *Blastocystis* sp. and *gp60* gene.

## Data Availability

All of the data used or analyzed during this study are available from the corresponding author on reasonable request. Representative nucleotide sequences have been deposited in GenBank, including *Cryptosporidium* spp., *gp60* (MT899227–MT899229), *G. duodenalis*, *E. bieneusi*, and *Blastocystis* sp.. Accession numbers of *Cryptosporidium* spp., *G. duodenalis*, *E. bieneusi* and *Blastocystis* sp. found in Tables 2, 3 and 4.

## Abbreviations

*SSU* rRNA: Small subunit rRNA
*gp60*: The 60 kDa glycoprotein gene
*gdh*: Glutamate dehydrogenase gene
CI: Confidence intervals
NJ: Neighbor-Joining
NHPs: Non-human primates
ITS: Internal transcribed spacer
