# Supplementary material for: Occurrence and molecular characterization of Cryptosporidium spp., Giardia duodenalis, Enterocytozoon bieneusi, and Blastocystis sp. in captive wild animals in zoos in Henan, China
Source: BMC Vet Res. 2021 Oct 18;17:332. doi: 10.1186/s12917-021-03035-0 (PMC8522229; doi:10.1186/s12917-021-03035-0)
Supplement: Supplementary file 2 — Additional file 2: Table S2. Nucleotide substitutions and indels at the ITS region of CHPM1 and CHDW1 genotypes. [file 12917_2021_3035_MOESM2_ESM.docx]

**Table S2.** Nucleotide substitutions and indels at the ITS region of CHPM1 and CHDW1 genotypes

| *E. bieneusi* genotype (Accession No.) | Nucleotide position | | | |
| --- | --- | --- | --- | --- |
| ITS | 78 | 89 | 107 | 111 |
| D (KX383624) | C | G | G | T |
| CHPM1 | T | - | - | - |
| CHWD1 | - | A | A | C |

Note: -: indicate identity to the reference sequence.
