# Supplementary material for: Occurrence and molecular characterization of Cryptosporidium spp., Giardia duodenalis, Enterocytozoon bieneusi, and Blastocystis sp. in captive wild animals in zoos in Henan, China
Source: BMC Vet Res. 2021 Oct 18;17:332. doi: 10.1186/s12917-021-03035-0 (PMC8522229; doi:10.1186/s12917-021-03035-0)
Supplement: Supplementary file 3 — Additional file 3: Table S3. Primers and reaction conditions used in the characterization of the SSU rRNA gene of Cryptosporidium spp., Giardia duodenalis, Enterocytozoon bieneusi, Blastocystis sp. and gp60 gene. [file 12917_2021_3035_MOESM3_ESM.docx]

**Table S3.** Primers used in the characterization of the *Cryptosporidium* spp., *Giardia duodenalis*, *Enterocytozoon bieneusi*, and *Blastocystis* sp.

| Gene | Nucleotide sequences of primer (5′- 3′) | Expected product size (bp) | Annealing temperature (ºC) | Reference |
| --- | --- | --- | --- | --- |
| SSU rRNA of *Cryptosporidium* spp. | SSU-F2: TTCTAGAGCTAATACATGCG | 840 | 55 | [45] |
|  | SSU-R2: CCCATTTCCTTCGAAACAGGA |  |  |  |
|  | SSU-F3: GGAAGGGTTGTATTTATTAGATAAAG |  | 55 |  |
|  | SSU-R4: CTCATAAGG TGCTGAAGGAGTA |  |  |  |
| *gdh* gene of Giardia dusodenalis | Gdh1: TTCCGTRTYCAGTACAACTC | 520 | 50 | [40] |
|  | Gdh2: ACCTCGTTCTGRGTGGCGCA |  |  |  |
|  | Gdh3: ATGACYGAGCTYCAGAGGCACGT |  | 50 |  |
|  | Gdh4: GTGGCGCARGGCATGATGCA |  |  |  |
| *ITS* gene of *E*. *bieneusi* | EBITS3: GATGGTCATAGGGATGAAGAGCTT | 392 | 57 | [42] |
|  | EBITS4: TATGCTTAAGTCCAGGGAG |  |  |  |
|  | EBITS1: AGGGATGAAGAGCTTCGGCTCTG |  | 50 |  |
|  | EBITS2.4: AGTGATCCTGTATTAGGGATATT |  |  |  |
| *Gp60* gene of *Cryptosporidium* spp. | AL3531: ATAGTCTCCGCTGTATTC | 400 | 55 | [41] |
|  | AL3533: AGATATATCTTGGTGCG |  |  |  |
|  | AL3532: TCCGCTGTATTCTCAGCC |  | 55 |  |
|  | LX0029: CGAACCACATTACAAATGAAGT |  |  |  |
| SSU rRNA of *Blastocystis* sp. | RD5: ATCTGGTTGATCCTGCCAGT | 600 | 55 | [43] |
|  | BhRDr: GAGCTTTTTAACTGCAACAACG |  |  |  |
